# Diagnosing chronic pancreatitis by endoscopic ultrasound assessing the association between ultrasound and pathological findings: A narrative review

**DOI:** 10.1002/deo2.164

**Published:** 2022-09-15

**Authors:** Akira Yamamiya, Atsushi Irisawa, Yoko Abe, Takahiro Arisaka, Toshihiko Ohnishi, Koki Hoshi, Tsunehiro Suzuki, Kazunori Nagashima, Ken Kashima, Yasuhito Kunogi, Fumi Sakuma, Koh Fukushi, Manabu Ishikawa, Nasuka Mizuguchi, Shintaro Yamaguchi, Keiichi Tominaga, Kenichi Goda

**Affiliations:** ^1^ Department of Gastroenterology Dokkyo Medical University School of Medicine Tochigi Japan

**Keywords:** chronic pancreatitis, endoscopic ultrasound, endoscopic ultrasound‐guided fine needle aspiration, pancreatic fibrosis, histopathological diagnosis

## Abstract

Endoscopic ultrasound (EUS) is widely recognized for its non‐invasiveness and for its usefulness in chronic pancreatitis (CP) diagnosis, including early CP. Although it is desirable to obtain a definitive diagnosis of CP by tissue sampling with EUS‐guided fine needle aspiration, histopathological changes in CP are heterogeneous in terms of the extent and the distribution of lesions. Therefore, histopathological diagnosis of appropriate tissue sampling by EUS‐fine needle aspiration is expected to be difficult. Furthermore, it is virtually impossible to match EUS images with pathological sections, making direct contrast between EUS findings and pathology difficult. This narrative review presents a discussion of the diagnosis of CP/early CP by EUS, particularly assessing the association between ultrasound and pathological findings. Recently, the histological corroboration and correlation of EUS findings related to CP have been clarified by surgical specimens, including those obtained from animal studies. Furthermore, remarkable advances have occurred in the objective and quantitative diagnosis of pancreatic fibrosis by EUS‐elastography. Future technological advances in EUS are expected to improve the accuracy of diagnosis of pancreatic fibrosis at earlier stages.

## INTRODUCTION

Chronic pancreatitis (CP), an irreversible and progressive inflammation of the pancreas, is characterized by extensive fibrosis of the pancreatic glands caused by persistent and recurrent inflammation, leading eventually to pancreatic exocrine and endocrine disorders.^1^, ^2^, ^3^ Several guidelines used for diagnosing CP recommend computed tomography (CT) and magnetic resonance imaging/cholangiopancreatography (MRI/MRCP) as the first imaging modalities. Endoscopic retrograde MRCP (ERCP) is also an important test for CP diagnosis, but when abnormalities occur in findings from such tests, many of them are non‐reversible CP.^4^, ^5^, ^6^, ^7^ Nevertheless, endoscopic ultrasound (EUS), which allows observation of the pancreas at close range with high resolution, has the potential to diagnose subtle changes, especially for cases of early CP (ECP) without calcification.^8^, ^9^, ^10^ Actually, EUS is widely recognized for its non‐invasiveness and for its usefulness in CP diagnosis. An important and persistent problem with EUS in ECP diagnosis is its lack of a histopathological gold standard. If EUS‐guided fine needle aspiration (EUS‐FNA) can perform the following observation with the easy and certain provision of tissue samples, then the problem described above is solvable. Histopathological changes in CP are heterogeneous in terms of the extent and the distribution of lesions. Therefore, histopathological diagnosis of appropriate tissue sampling by EUS‐FNA is expected to be difficult. Furthermore, it is virtually impossible to match EUS images with pathological sections, making direct contrast between EUS findings and pathology difficult. Consequently, if the diagnostic imaging of CP using EUS confirms the histological findings clearly, then the importance of EUS for the diagnosis of ECP can be confirmed. This narrative review was conducted to describe the diagnosis of CP/ECP using EUS, assessing the association between ultrasound and pathological findings. First, we discussed the diagnostic methods and accuracy of CP by EUS. Second, we summarized the progress of EUS diagnosis for CP, particularly addressing the association between EUS and pathological findings in CP, including those obtained from animal studies.

## OUTLINE OF PROGRESS IN DIAGNOSING CP BY EUS

**FIGURE 1:**
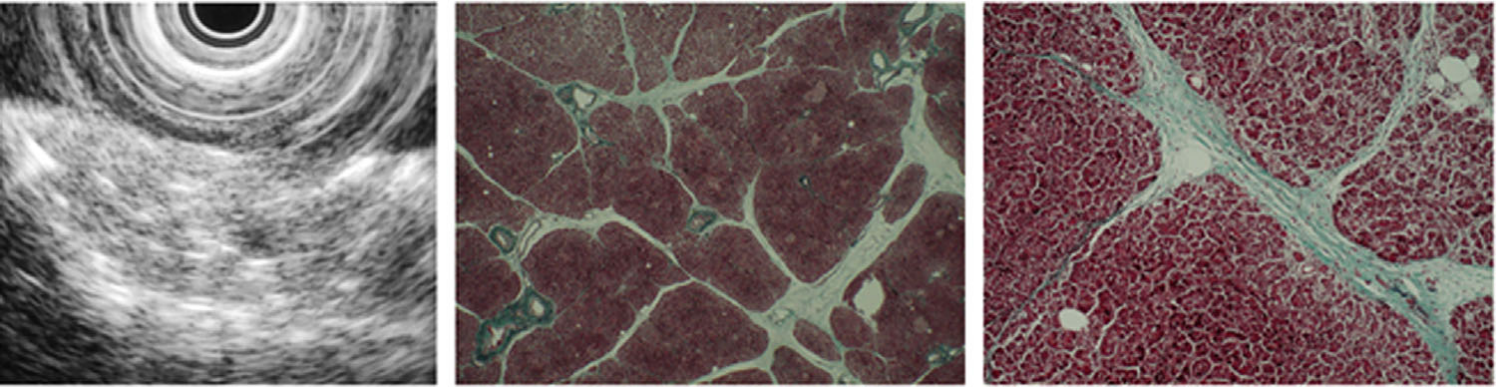
Underwater endoscopic ultrasound findings of hyperechoic strands and autopsy histological findings of bridging fibrosis

In 1992, Zuccaro et al. were the first to report EUS as useful to assess parenchymal and ductal images of the pancreas. Moreover, they were the first to report its usefulness for diagnosing CP.^11^ Reports describing the usefulness of EUS for diagnosing CP were published thereafter. Many reported studies compared EUS findings with pancreatic ductal findings on ERCP and described EUS findings were consistent with endoscopic retrograde pancreatography (ERP) findings in more than 80% of cases.^12^, ^13^, ^14^, ^15^, ^16^, ^17^, ^18^, ^19^ Wallace et al. reported that EUS findings such as hyperechoic foci, hyperechoic strands, lobularity, and hyperechoic ductal margins are consistent with histological findings indicating fibrosis of the parenchyma, including focal fibrosis, bridging fibrosis, interlobular fibrosis and periductal fibrosis^8^, ^12^, ^18^ (Table 1 and Figure 1). Regarding the correlation between MRCP and histological findings in CP, the usefulness of secretin‐enhanced MRCP (S‐MRCP) was assessed by Zhang et al.^20^ Reportedly, S‐MRCP parameters are correlated with the histopathological severity of CP. Based on another report, Souza et al. evaluated and confirmed the high diagnostic accuracy of an S‐MRCP CP severity index for diagnosing CP using EUS based on the Rosemont criteria.^21^

**TABLE 1:** Association between endoscopic ultrasound (EUS) findings and histological findings for chronic pancreatitis

EUS findings^8^, ^12^, ^18^	Presumable histological findings^8^, ^12^, ^18^
Hyperechoic foci	Focal fibrosis
Hyperechoic strands	Bridging fibrosis
Lobular out gland margin	Fibrosis, glandular atrophy
Lobularity	Interlobular fibrosis
Cyst	Cysts/pseudocysts
Stone	Calcified stones in the duct
Calcification	Parenchymal calcification
Main duct dilation	>2.4 mm in the head, >1.8 mm in the body, >1.2 mm in the tail
Dilated side branches	Dilated side branches
Duct irregularity	Focal dilation/narrowing
Hyperechoic duct margins	Periductal fibrosis
Atrophy	Atrophy
Non‐homogeneous echo pattern	Edema

Pungpapong et al. compared findings obtained using EUS and MRCP in CP.^22^ The sensitivity of EUS was higher than that of MRCP, although the specificities of EUS and MRCP were similar. Furthermore, they reported a sensitivity of 98% when either EUS or MRCP was abnormal, and reported a specificity of 100% when both were abnormal. The combination of EUS and MRCP has reportedly increased the diagnostic accuracy in ECP.^23^ Together, MRCP and EUS might replace ERCP for diagnosing CP. Consequently, recent reports have demonstrated MRCP as useful as an imaging test to reflect the histological findings of CP, but EUS has even higher diagnostic accuracy.

In light of the points raised in the discussion above, EUS has been recognized as a modality that should be used actively for diagnosing CP, especially fibrous changes in the parenchyma and ductal wall.

## EUS IMAGE OF NORMAL PANCREAS

Normal pancreatic parenchyma is shown by EUS as a fine reticular pattern. No dilated or tortuous main or dilated side branch is observed within the pancreatic parenchymal echogenicity.^24^ The main pancreatic duct (MPD) wall is observed to have a homogeneous linear echo, although it is slightly hyperechoic, with a 2.4 mm diameter at the head, 1.8 mm at the body, and about 1.2 mm at the tail.^25^ Generally speaking, the EUS findings of CP are defined on these bases.

## DIAGNOSIS OF CP USING EUS

**FIGURE 2:**
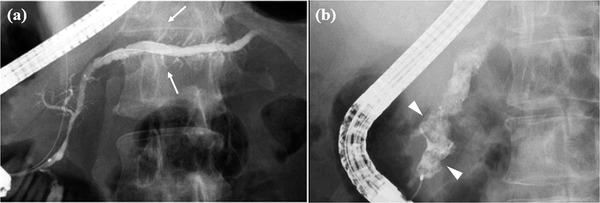
Endoscopic retrograde pancreatography findings of Cambridge classification for chronic pancreatitis: (a) Abnormal branches. (b) Intraductal filling defects or calculi

**FIGURE 3:**
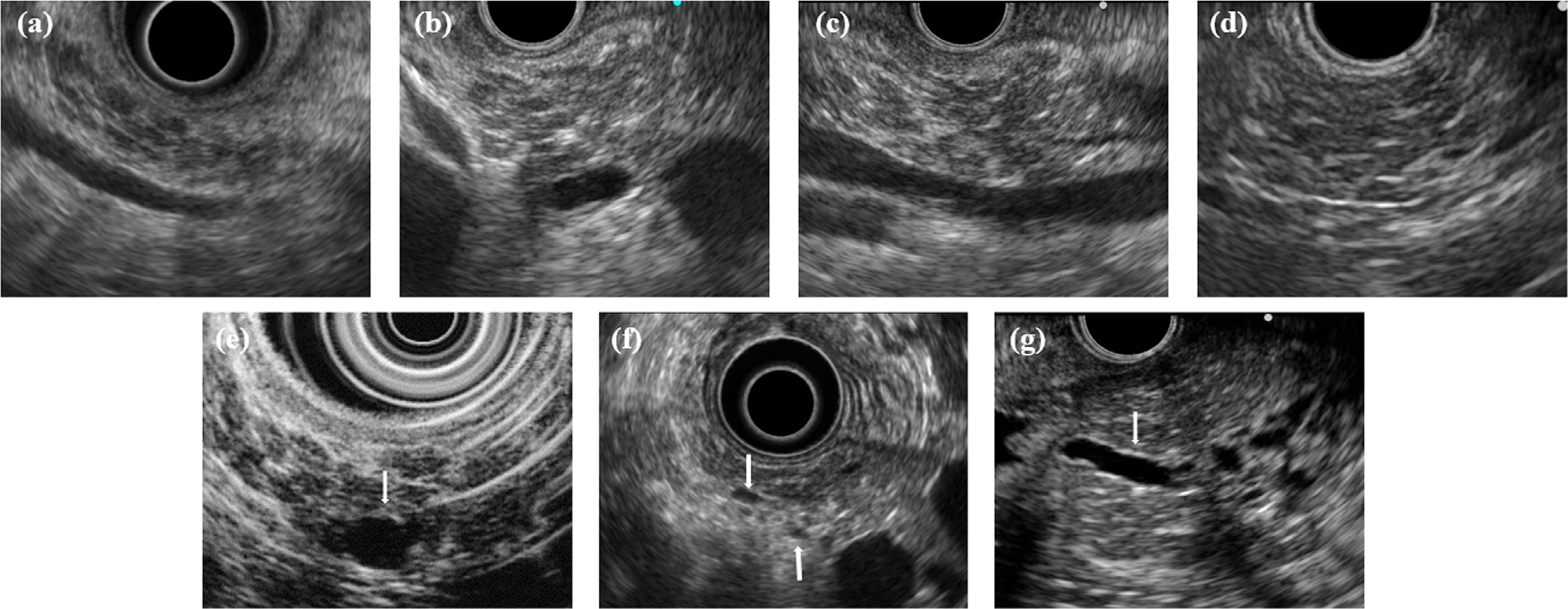
Endoscopic ultrasound findings of Rosemont classification for chronic pancreatitis: (a) Lobularity with honeycombing. (b) Lobularity without honeycombing. (c) Hyperechoic foci without shadowing. (d) Strands. (e) Cysts. (f) Dilated side branches. (g) Hyperechoic main pancreatic duct margin

Evaluation of the diagnosis and severity classification of CP by EUS demonstrated two criteria: 1) the total number of EUS finding based on ERP findings (Cambridge classification) as the gold standard (Table S1 and Figure 2)^15^, ^24^, ^26^, ^27^; 2) classification considering the weight of each finding, the Rosemont classification (Table S2 and Figure 3).^28^ Regarding classification using the number of EUS findings, 2–4 findings are generally considered ‘mild’, 5–6 findings are regarded as ‘moderate’, and more than 7 findings are considered ‘severe’. EUS has been shown to have an agreement rate of approximately 80% with the ERCP diagnosis. Irisawa et al. conducted a similar study of patients who had undergone ERCP and EUS.^17^, ^29^ They reported that more than 80% of patients with ‘borderline’ or higher changes in ERCP classification had three or more EUS findings. Further detailed analysis revealed that 3–4 cases were in agreement with the ERCP classification of mild, 5–6 cases were in agreement with moderate, and more than seven cases were in agreement with severe, with an agreement rate exceeding 80%. These results indicate that, as reported earlier, EUS is useful not only for diagnosing the presence of CP but also for classifying severity.

The Rosemont classification, which considers the weighting of the respective findings,^28^ was proposed in 2009. The classification is now commonly used as a diagnostic method for CP by EUS. Specifically, it includes major A for hyperechoic foci with shadowing and MPD calculi and hyperechoic foci with shadowing, and major B for lobularity with honeycombing, with eight other items classified as minor. Points are allocated according to these ratings, which are classified into four levels: (1) consistent with CP, (2) suggestive of CP, (3) indeterminate for CP, and (4) normal. Although the Rosemont classification is a diagnostic method classified by severity and although it is not classified in chronological order, ‘indeterminate’ in the Rosemont classification is regarded as corresponding to ECP.

Some reports have compared the diagnostic performance of conventional methods with that of the Rosemont classification, but no significant differences in the correct diagnosis rates are clear, partly because of interobserver reliability (IOR) and the complexity of the Rosemont classification.^24^, ^25^, ^26^, ^27^, ^30^, ^31^

In recent years, EUS‐elastography (EUS‐EG) has become available, further improving the ability of EUS to diagnose fibrosis in CP.^32^, ^33^, ^34^, ^35^, ^36^, ^37^, ^38^, ^39^, ^40^ Actually, EUS‐EG is a more objective method to diagnose fibrosis of the pancreas using EUS.^41^ This is a new diagnostic technique for measuring tissue elasticity (stiffness) by application of vibrational energy to the tissue externally, such as manual compression or heartbeat, and for measuring the resulting strain and waves. Giovannini et al. first reported the elastic score: a color pattern diagnosis. The elastic score, color pattern, and heterogenicity of distribution of the EG were classified into five types.^42^, ^43^ Generally speaking, CP, which has a higher degree of hardness than a normal pancreas, appears as blue and heterogeneous on EUS‐EG as the disease progresses, which correlates with the Rosemont classification.^27^, ^32^, ^44^ Currently, the usefulness of EUS‐strain EG (EUS‐SE) and of EUS‐shear wave EG (EUS‐SWE) have been reported.^40^

## COMPARISON OF EUS FINDINGS WITH HISTOPATHOLOGICAL FINDINGS

The contrast between EUS and histopathology findings is an important consideration for the diagnosis of CP by EUS. Because obtaining tissue samples in ECP is difficult, several comparative histopathological findings of CP, including animal studies, have been reported (Table 2).

**TABLE 2:** Comparison between endoscopic ultrasound findings and histological findings for chronic pancreatitis

	Year	Number	Tissue sampling method	EUS criteria	Results	Correlation
Chong^19^	2007	41 (CP^†^)	Operation	Conventional	≥3 EUS criteria Sensitivity 83%, Specificity 80%	Weak (*r* = 0.4, *p* = 0.01)
Varadarajulu^45^	2007	42 (tumor40, CP^†^2)	Operation	Conventional	≥4 EUS criteria Sensitivity 91%, Specificity 86%	Strong (*r* = 0.85, *p* = 0.0001)
Albashir^49^	2010	23 (CP^†^)	Operation	Conventional	≥4 EUS criteria Sensitivity 84%, Specificity 100%	Strong (*r* = 0.72, *p* < 0.01)
Leblanc^46^	2014	100 (CP+IPMN)	Operation	Rosemont	Ph: ≥3 EUS criteria Sensitivity 54%, Specificity 22% Pb‐t: ≥3 EUS criteria Sensitivity 54%, Specificity 22%	Weak (*r* = 0.33, *p* < 0.05)
Trikudanathan^47^	2016	68 (CP^†^)	Operation	Conventional	≥4 EUS criteria Sensitivity 61%, Specificity 75%	Weak (*r* = 0.2, *p* < 0.05)
Trikudanathan^48^	2017	50 (CP)	Operation	Conventional+ Rosemont	15/26 (96%) Reportive 12/15 (80%) Indeterminate 5/9 (56%) Normal	FS ≥ 2

Abbreviations: CP, chronic pancreatitis; EUS, endoscopic ultrasonography; FS, fibrosis score; IPMN, intraductal papillary mucinous neoplasm; Ph, pancreas head; Pb‐t, pancreas body‐tail.

In many studies, the fibrosis score (FS) proposed by Ammann et al. has been used to assess fibrosis.^18^ The evaluation method first assesses whether perilobular fibrosis is focal or diffuse; then it classifies the perilobular fibrosis into one of three levels: mild, moderate, and severe. The score is then assigned from 0 for no fibrosis, 1 for focal–mild to 6 for diffuse–severe. Intralobular fibrosis (interlobular) is then similarly scored 0–6 points. The two are combined for overall evaluation (range 0–12 points). Most reports of EUS versus histopathological findings define CP as a total of two or more points of FS.

All related reports describe that the best balance of sensitivity and specificity was found when three, four, or more EUS findings were obtained. Of the studies which have examined correlation, only two indicated a correlation coefficient (*r*) of 0.7 or more. Chong et al. reported the median FS in CP was 7 and reported that if three or more EUS findings were present, then the patient could be regarded as having fibrosis histologically.^19^ The sensitivity and specificity were best balanced, respectively, at 83% and 80%. The correlation was weak but significant. Nevertheless, no correlation between individual EUS findings and FS was found.

Varadarajulu et al. prospectively studied the contrast between EUS and histopathological findings in 40 cases of pancreatic tumors, including 29 cases of pancreatic cancer and 2 cases of CP in surgical specimens.^45^ A good balance with moderate fibrosis with FS≥6 was reported with a sensitivity of 91% and specificity of 86% for ≥4 EUS findings. Although no one‐to‐one correspondence was found between individual EUS findings, partial fibrosis and fibrosis around the pancreatic ducts were observed in areas where mild foci and strands were present. In areas of marked lobularity (corresponding to lobularity with honeycombing in the Rosemont classification), cirrhosis‐like marked fibrosis was observed within and between lobes and inflammatory cell infiltration.

ECP was also investigated by comparing EUS findings and resection specimens. With hyperechoic foci, hyperechoic strands and lobulations in the pancreatic parenchyma, and dilated or irregular MPDs, side branch dilation, and hyperechoic ductal margins in the pancreatic ducts were all consistent with tissue findings. This study is particularly important because it is based on ECP without calcification. It is regarded as an accurate representation of the objectivity of EUS findings.

LeBlanc et al. classified EUS findings of the pancreas head in FS into one of three levels: 1–4 points as mild, 5–8 points as moderate, and 9–12 points as severe.^46^ Among these, three or more EUS findings in the pancreatic head are regarded as indicating moderate fibrosis. In cases with severe fibrosis, EUS findings of lobularity with honeycombing, hyperechoic foci with/without shadowing, MPD dilatation, main duct irregularity, and branching duct dilatation were associated with pathological findings. Moreover, the MPD findings were assumed to reflect fibrosis of the pancreatic parenchyma around the pancreatic duct.

Trikudanathan et al. compared wedge biopsy of the pancreas head–body–tail with EUS findings in patients who underwent total pancreatectomy plus autologous islet transplantation.^47^ Four or more EUS findings were assessed as the threshold, but no satisfactory correlation with histopathological findings was obtained. The presence of fibrosis in pathological findings in cases with two or more EUS items, which is usually considered normal, was also examined; the sensitivity was reported to be 83%. Furthermore, they stated that EUS findings of fewer than two items do not indicate a normal pancreas without fibrosis. Trikudanathan et al. conducted a similar study using the Rosemont classification,^48^ which obtained findings suggesting that CP can be a predictor of CP, but showing that the correlation between EUS findings and the degree of fibrosis was weak. Specifically, 5/9 cases were diagnosed as having FS 2 or more, that is, fibrosis, despite normal Rosemont classification. This finding reflects the difficulty in diagnosing a normal pancreas even when using EUS, which is regarded as having the best resolution for pancreatic observation.

Albashir et al. also reported a significant correlation between EUS findings and histopathology findings in surgical cases.^49^ The diagnostic performance of EUS for CP based on histological findings was 84% sensitivity and 100% specificity, according to this study.

Bhutani et al. performed pathological autopsies on patients diagnosed with CP by EUS performed before death. They particularly examined pancreatic tissue characteristics.^50^ In 10 out of 11 cases where pancreatic tissue was identifiable without autolysis, pathological findings of CP were also found in the pancreas at pathological autopsy. Bhutani et al. created a CP model by inserting a pancreatic duct stent in dogs and implanting it for 4 weeks.^51^ Then, the pancreatic parenchyma was observed using EUS before and after. The EUS findings not seen before stent placement (lobularity, hyperechoic and hypoechoic foci, increased echogenic septations, visible pancreatic duct side branches, and irregular margins of the MPD) were identified 2–4 weeks after stenting. Histological examination during the same period showed findings of CP. The study yielded valuable findings for EUS observations indicating the progression of CP. The findings strongly demonstrate the objectivity of EUS findings for the diagnosis of CP.

Some reports have described examinations of whether high‐echo or low‐echo areas in EUS findings reflect actual fibrosis. Okabe et al. compared tissue specimens and EUS findings for patients who underwent EUS before and after steroid treatment for autoimmune pancreatitis and who underwent surgery because malignancy could not be ruled out despite steroid treatment.^52^ They reported that the high‐echoic areas of lobularity were infiltrated by inflammatory cells, whereas the internal hypoechoic areas were fibrosis. Sekine et al. contrasted and examined EUS findings and pathology findings in diagnostic criteria for ECP 2019 in Japan (DCECP2019)^7^ from surgical specimens.^53^ The results demonstrated that lobularity in EUS reflected inflammatory cell infiltration, atrophy, and fibrosis of the pancreatic adenocytes. Hyperechoic MPD margin reflected thinning of the duct wall in pathological findings.

Recent reports have described the usefulness of EUS‐EG for diagnosing pancreatic fibrosis. Yamashita et al. assessed the utility of EUS‐SWE for CP diagnosis and pancreatic fibrosis^54^ and found that shear‐wave velocity (*V*s) has a significant and positive correlation with the Rosemont classification and several EUS features of CP. The EUS‐SWE results were consistent with CP (*V*s 2.98 m/s) and were suggestive of CP (*V*s 2.95 m/s). The results were significantly higher than those found for normal tissue (*V*s 1.52 m/s). Actually, EUS‐SWE also showed high accuracy for diagnosing CP, with the area under the receiver operating characteristic curve of 0.97. The *V*s cut‐off of 2.19 m/s showed 100% sensitivity and 94% specificity when diagnosing CP. Collectively, the results imply that EUS‐EG is capable of quantifying fibrosis in CP. Itoh et al. conducted this study using tissue specimens.^32^ They classified the degree of fibrosis of the tissue on the head side of the pancreas in 58 surgical cases into four levels, from normal to severe, and examined histograms of the EG using special software. Of the four parameters (mean, standard deviation, skewness, and kurtosis), the mean (mean value of elasticity) showed the best negative correlation with pancreatic fibrosis (*r* = −0.75). Although the instability of measurements remains a future challenge, EUS‐EG might help to estimate pathological fibrosis in CP/ECP.

Results show that EUS including EUS‐EG can be very useful for assessing CP. Reports describing the correlation between EUS findings and pathological findings are beginning to be published. On the other hand, the sensitivity and specificity of individual EUS findings in the diagnosis of CP have not yet been reported. This has not yet been reported for the animal model as well.^15^ However, it has already been shown that hyperechoic foci and hyperechoic strands are of diagnostic importance in mild CP, main duct dilatation, and dilated side branches in moderate CP, and calcification in addition to findings in moderate CP in severe CP.^17^ Besides, although not in terms of histological correlation with EUS findings, hyperechoic foci, MPD calculi, lobularity, strands, MPD contour, dilated side branches and hyperechoic MPD margin have been reported to correlate with risk factors for CP in the mechanistic definition, such as ethanol intake, smoking status, and/or history of acute pancreatitis.^3^, ^55^ From this point of view, individual EUS findings of CP are important. In Japan, the diagnostic criteria for CP/ECP were revised in 2019 (DCCP/ECP2019). To solve the problem of IOR in EUS findings for ECP, two of the following four criteria were required: (1) Hyperechoic foci; non‐shadowing/Stranding, (2) Lobularity, (3) Hyperechoic MPD margin, and (4) Dilated side branches. We analyzed the changes in EUS findings with DCECP2019 and examined the validity of the revision.^31^ The overall concordance rate of EUS findings in the old criteria in 2009 (DCECP2009) was *K*‐value = 0.424, and the overall diagnostic concordance rate of EUS findings in DCECP 2019 was *K‐*value = 0.618. DCECP2019 combines EUS findings that were similar to DCECP2009. This point contributed to the increase in IOR and the concordance rate of EUS diagnostic ability. Thus, the revision of DCECP 2019 is expected to further improve diagnostic ability. Additional studies must be conducted in the future to assess the utility of these methods.

## Conclusions

As these reports indicate, the histological corroboration and correlation of EUS findings related to CP have been clarified in recent years. Particularly, the relationship between pathological findings and EUS findings in ECP will become increasingly important in terms of early diagnosis. When CP/ECP is assessed by EUS, it is important to compare each EUS finding with the presumed histological findings. The process might allow CP/ECP stage to be inferred without pathological examination. Several issues have been proposed, such as the problem of IOR in EUS findings, appropriate tissue assessment methods, and the difficulty of pancreatic fibrosis related to aging or diabetes mellitus. These points complicate the diagnosis of fibrosis in CP by EUS. In any case, the specific pathology of individual EUS findings in the diagnosis of CP demands further investigation.

## CONFLICT OF INTEREST

The authors declare that they have no conflict of interest.

## FUNDING INFORMATION

None.

## Supporting information


**Table S1** ERP findings of Cambridge classification for chronic pancreatitis
**Table S2** Rosemont classification for chronic pancreatitis

## References

[1] Witt H , Apte MV , Keim V , Wilson JS . Chronic pancreatitis: Challenges and advances in pathogenesis, genetics, diagnosis, and therapy. Gastroenterology 2007; 132: 1557–73. 17466744 10.1053/j.gastro.2007.03.001

[2] Braganza JM , Lee SH , McCloy RF , McMahon MJ . Chronic pancreatitis. Lancet 2011; 377: 1184–97. 21397320 10.1016/S0140-6736(10)61852-1

[3] Whitcomb D.C , Frulloni L , Garg P *et al*. Chronic pancreatitis: An international draft consensus proposal for a new mechanistic definition. Pancreatology 2016; 16: 218–24. 26924663 10.1016/j.pan.2016.02.001 PMC6042966

[4] Masamune A , Kikuta K , Kume K *et al*. Nationwide epidemiological survey of chronic pancreatitis in Japan: Introduction and validation of the new Japanese diagnostic criteria 2019. J Gastroenterol 2020; 55: 1062–71. 32676800 10.1007/s00535-020-01704-9

[5] Masamune A , Nabeshima T , Kikuta K *et al*. Prospective study of early chronic pancreatitis diagnosed based on the Japanese diagnostic criteria. J Gastroenterol 2019; 54: 928–35. 31270692 10.1007/s00535-019-01602-9

[6] Masamune A , Kikuta K , Nabeshima T *et al*. Nationwide epidemiological survey of early chronic pancreatitis in Japan. J Gastroenterol 2017; 52: 992–1000. 28130705 10.1007/s00535-017-1311-8

[7] Masamune A , Irisawa A , Kikuta K *et al*. Background and summary of the clinical diagnostic criteria for chronic pancreatitis 2019 [in Japanese, English abstract]. J Jpn Pancreas Soc 2019; 34: 279–81.

[8] DeWitt J , McGreevy K , LeBlanc J , McHenry L , Cummings O , Sherman S . EUS‐guided trucut biopsy of suspected nonfocal chronic pancreatitis. Gastrointest Endosc 2005; 62: 76–84. 15990823 10.1016/s0016-5107(05)00504-3

[9] Raimondo M , Wallance MB . Diagnosis of early chronic pancreatitis by endoscopic ultrasound. Are we there yet? JOP 2004; 5: 1–7. 14730117

[10] Kuwahara T , Hirooka Y , Kawashima H *et al*. Quantitative diagnosis of chronic pancreatitis using EUS elastography. J Gastroenterol 2017; 52: 868–74. 27995327 10.1007/s00535-016-1296-8

[11] Zuccaro G Jr , Sivak MV Jr . Endoscopic ultrasound in the diagnosis of chronic pancreatitis. Endoscopy 1992; 24: 347–9. 1633779 10.1055/s-2007-1010497

[12] Wallace MB , Hawes RH . Endoscopic ultrasound in the evaluation and treatment of chronic pancreatitis. Pancreas 2001; 23: 26–35. 11451144 10.1097/00006676-200107000-00004

[13] Nattermann C , Goldschmidt AJ , Dancygier H . Endosonography in chronic pancreatitis – A comparison between endoscopic retrograde pancreatography and endoscopic ultrasonography. Endoscopy 1993; 25: 565–70. 8119205 10.1055/s-2007-1010406

[14] Buscail L , Escourrou J , Moreau J *et al*. Endoscopic ultrasonography in chronic pancreatitis: A comparative prospective study with conventional ultrasonography, computed tomography, and ERCP. Pancreas 1995; 10: 251–7. 7624302

[15] Catalano MF , Lahoti S , Geenen JE , Hogan WJ . Prospective evaluation of endoscopic ultrasonography, endoscopic retrograde pancreatography, and secretin test in the diagnosis of chronic pancreatitis. Gastrointest Endosc 1998; 48: 11–7. 9684658 10.1016/s0016-5107(98)70122-1

[16] Sahai AV , Zimmerman M , Aabakken L *et al*. Prospective assessment of the ability of endoscopic ultrasound to diagnose, exclude, or establish the severity of chronic pancreatitis found by endoscopic retrograde cholangiopancreatography. Gastrointest Endosc 1998; 48: 18–25. 9684659 10.1016/s0016-5107(98)70123-3

[17] Irisawa A , Katakura K , Ohira H *et al*. Usefulness of endoscopic ultrasound to diagnose the severity of chronic pancreatitis. J Gastroenterol 2007; 42: 90–4. 17238035 10.1007/s00535-006-1916-9

[18] Ammann RW , Heitz PU , Klöppel G . Course of alcoholic chronic pancreatitis: A prospective clinicomorphological long‐term study. Gastroenterology 1996; 111: 224–31. 8698203 10.1053/gast.1996.v111.pm8698203

[19] Chong AK , Hawes RH , Hoffman BJ , Adams DB , Lewin DN , Romagnuolo J . Diagnostic performance of EUS for chronic pancreatitis: A comparison with histopathology. Gastrointest Endosc 2007; 65: 808–14. 17466199 10.1016/j.gie.2006.09.026

[20] Zhang TT , Wang L , Wang DB , Huang ZJ , Li YH , Lu JP . Correlation between secretin‐enhanced MRCP findings and histopathologic severity of chronic pancreatitis in a cat model. Pancreatology 2013; 13: 491–7. 24075513 10.1016/j.pan.2013.08.003

[21] Souza D , Alessandrino F , Ketwaroo G.A , Sawhney M , Mortele KJ . Accuracy of a novel noninvasive secretin‐enhanced MRCP severity index scoring system for diagnosis of chronic pancreatitis: Correlation with EUS‐based Rosemont criteria. Radiol Med 2020; 125: 816–26. 32266691 10.1007/s11547-020-01181-3

[22] Pungpapong S , Wallace MB , Woodward TA , Noh KW , Raimondo M . Accuracy of endoscopic ultrasonography and magnetic resonance cholangiopancreatography for the diagnosis of chronic pancreatitis: A prospective comparison study. J Clin Gastroenterol 2007; 41: 88–93. 17198070 10.1097/MCG.0b013e31802dfde6

[23] Ito T , Ikeura T , Tanaka T *et al*. Magnetic resonance cholangiopancreatography findings in early chronic pancreatitis diagnosed according to the Japanese Diagnostic Criteria. Pancreatology 2020; 20: 596–601. 32371200 10.1016/j.pan.2020.04.008

[24] Sahai AV , Zimmerman M , Aabakken L *et al*. Prospective assessment of the ability of endoscopic ultrasound to diagnose, exclude, or establish the severity of chronic pancreatitis found by endoscopic retrograde cholangiopancreatography. Gastrointest Endosc 1998; 8: 18–25. 10.1016/s0016-5107(98)70123-3 9684659

[25] Wiersema MJ , Hawes RH , Lehman GA , Kochman ML , Sherman S , Kopecky KK . Prospective evaluation of endoscopic ultrasonography and endoscopic retrograde cholangiopancreatography in patients with chronic abdominal pain of suspected pancreatic origin. Endoscopy 1993; 25: 555–64. 8119204 10.1055/s-2007-1010405

[26] Sarner M , Cotton PB . Classification of pancreatitis. Gut 1984; 25: 756–9. 6735257 10.1136/gut.25.7.756 PMC1432589

[27] Bhutani MS . Endoscopic ultrasound in pancreatic diseases. Indications, limitations, and the future. Gastroenterol Clin North Am 1999; 28: 747–70. 10503148 10.1016/s0889-8553(05)70085-6

[28] Catalano M.F , Sahai A , Levy M *et al*. EUS‐based criteria for the diagnosis of chronic pancreatitis: The Rosemont classification. Gastrointest Endosc 2009; 69: 1251–61. 19243769 10.1016/j.gie.2008.07.043

[29] Katakura K . Evaluation of the ability of endoscopic ultrasound to diagnose early chronic pancreatitis. Gastrointest Endosc 2002; 6: S116.

[30] Yamamiya A , Irisawa A , Kashima K *et al*. Interobserver reliability of endoscopic ultrasonography: Literature review. Diagnostics 2020; 10: 953. 10.3390/diagnostics10110953 PMC7696989 33203069

[31] Yamamiya A , Irisawa A , Tominaga K *et al*. Interobserver reliability of the endoscopic ultrasound criteria for the diagnosis of early chronic pancreatitis: Comparison between the 2009 and 2019 Japanese Diagnostic Criteria. Diagnostics 2021; 11: 431. 33802623 10.3390/diagnostics11030431 PMC8000630

[32] Hirooka Y , Kuwahara T , Irisawa A *et al*. JSUM ultrasound elastography practice guidelines: Pancreas. J Med Ultrason 2001; 42: 151–74. 10.1007/s10396-014-0571-7 26576568

[33] Yamashita Y , Kitano M . Benefits and limitations of each type of endoscopic ultrasonography elastography technology for diagnosis of pancreatic diseases. Dig Endosc 2021; 33: 554–6. 33210343 10.1111/den.13870

[34] Sato A , Irisawa A , Bhutani MS *et al*. Significance of normal appearance on endoscopic ultrasonography in the diagnosis of early chronic pancreatitis. Endosc Ultrasound 2018; 7: 110–8. 28685746 10.4103/2303-9027.209870 PMC5914182

[35] Ohno E , Hirooka Y , Kawashima H *et al*. Feasibility and usefulness of endoscopic ultrasonography‐guided shear‐wave measurement for assessment of autoimmune pancreatitis activity: A prospective exploratory study. J Med Ultrason 2019; 46: 425–33. 10.1007/s10396-019-00944-4 PMC6765472 30993580

[36] Ohno E , Hirooka Y , Kawashilna H , Ishikawa T . Feasibility of EUS‐guided shear‐wave measurement: A preliminary clinical study. Endosc Ultrasound 2019; 8: 215–6. 30924448 10.4103/eus.eus_6_19 PMC6589995

[37] Yamashita Y , Tanioka K , Kawaji Y *et al*. Utility of elastography with endoscopic ultrasonography shear‐wave measurement for diagnosing chronic pancreatitis. Gut Liver 2020; 14: 659–64. 31722469 10.5009/gnl19170 PMC7492489

[38] Ohno E , Kawashima H , Ishikawa T *et al*. Diagnostic performance of endoscopic ultrasonography‐guided elastography for solid pancreatic lesions: Shear‐wave measurements versus strain elastography with histogram analysis. Dig Endosc 2021; 33: 629–38. 32662150 10.1111/den.13791

[39] Yamashita Y , Tanioka K , Kawaji Y *et al*. Endoscopic ultrasonography shear wave as a predictive factor of endocrine/exocrine dysfunction in chronic pancreatitis. J Gastroenterol Hepatol 2021; 36: 391–6. 32511808 10.1111/jgh.15137

[40] Yamashita Y , Kitano M . Benefits and limitations of each type of endoscopic ultrasonography elastography technology for diagnosis of pancreatic diseases. Dig Endosc 2021; 33: 554–6. 33210343 10.1111/den.13870

[41] Yamamiya A , Irisawa A , Hoshi K *et al*. Recent Advances in endosonography–elastography: Literature review. J Clin Med 2021; 10: 3739. 34442035 10.3390/jcm10163739 PMC8397158

[42] Giovannini M , Hookey LC , Bories E , Pesenti C , Monges G , Delpero JR . Endoscopic ultrasound elastography: The first step towards virtual biopsy? Preliminary results in 49 patients. Endoscopy 2006; 38: 344–8. 16680632 10.1055/s-2006-925158

[43] Kuwahara T , Hirooka Y , Kawashima H *et al*. Quantitative diagnosis of chronic pancreatitis using EUS elastography. J Gastroenterol 2017; 52: 868–74. 27995327 10.1007/s00535-016-1296-8

[44] Itoh Y , Itoh A , Hirooka Y *et al*. Quantitative analysis of diagnosing pancreatic fibrosis using EUS‐elastography (comparison with surgical specimens). J Gastroenterol 2014; 49: 1183–92. 24026103 10.1007/s00535-013-0880-4

[45] Varadarajulu S , Eltoum I , Tamhane A , Eloubeidi MA . Histopathologic correlates of noncalcific chronic pancreatitis by EUS: A prospective tissue characterization study. Gastrointest. Endosc 2007; 66: 501–9. 17640639 10.1016/j.gie.2006.12.043

[46] LeBlanc JK , Chen JH , Al‐Haddad M *et al*. Endoscopic ultrasound and histology in chronic pancreatitis: How are they associated? Pancreas 2014; 43: 440–4. 24622076 10.1097/MPA.0000000000000047

[47] Trikudanathan G , Vega‐Peralta J , Mallli A *et al*. Diagnostic performance of endoscopic ultrasound (EUS) for non‐calcific chronic pancreatitis (NCCP) based on histopathology. Am J Gastroenterol 2016; 111: 568–74. 26952577 10.1038/ajg.2016.48

[48] Trikudanathan G , Munigala S , Barlass U *et al*. Evaluation of Rosemont criteria for non‐calcific chronic pancreatitis (NCCP) based on histopathology – A retrospective study. Pancreatology 2017; 17: 63–9. 27836330 10.1016/j.pan.2016.10.010

[49] Albashir S , Bronner MP , Parsi MA , Walsh RM , Stevens T . Endoscopic ultrasound, secretin endoscopic pancreatic function test, and histology: Correlation in chronic pancreatitis. Am J Gastroenterol 2010; 105: 2498–503. 20606675 10.1038/ajg.2010.274

[50] Bhutani MS , Arantes VN , Verma D *et al*. Histopathologic correlation of endoscopic ultrasound findings of chronic pancreatitis in human autopsies. Pancreas 2009; 38: 820–4. 19657310 10.1097/MPA.0b013e3181b2bc1a

[51] Bhutani MS , Ahmed I , Verma D , Xiao SY , Brining D . An animal model for studying endoscopic ultrasound changes of early chronic pancreatitis with histologic correlation: A pilot study. Endoscopy 2009; 41: 352–6. 19340741 10.1055/s-0029-1214492

[52] Okabe Y , Ishida Y , Kaji R *et al*. Endoscopic ultrasonographic study of autoimmune pancreatitis and the effect of steroid therapy. J Hepatobiliary Pancreat Sci 2012; 19: 266–73. 21671062 10.1007/s00534-011-0392-7

[53] Sekine M , Tanaka A , Akimoto M *et al*. A comparative study of endoscopic ultrasonography and histopathology images for the diagnosis of early chronic pancreatitis. Pancreas 2021; 50: 1173–9. 34714281 10.1097/MPA.0000000000001893 PMC8565505

[54] Yamashita Y , Tanioka K , Kawaji Y *et al*. Utility of elastography with endoscopic ultrasonography shear‐wave measurement for diagnosing chronic pancreatitis. Gut Liver 2020; 14: 659–64 31722469 10.5009/gnl19170 PMC7492489

[55] Yamabe A , Irisawa A , Bhutani MS *et al*. Validity of endoscopic ultrasound findings of chronic pancreatitis: Evaluation from the viewpoint of disease risk factors. Digestion 2021; 102: 289–97. 31801132 10.1159/000504780

