# Supplementary material for: Diagnosing chronic pancreatitis by endoscopic ultrasound assessing the association between ultrasound and pathological findings: A narrative review
Source: DEN Open. 2022 Sep 15;3(1):e164. doi: 10.1002/deo2.164 (PMC9478231; doi:10.1002/deo2.164)
Supplement: Supplementary file 1 — Table S1 ERP findings of Cambridge classification for chronic pancreatitis Table S2 Rosemont classification for chronic pancreatitis [file DEO2-3-e164-s001.docx]

**Supplementary Table 1.** **ERP findings of Cambridge classification for chronic pancreatitis**

|  | **ERP**^†^ **findings** |
| --- | --- |
| Normal | Quality study visualizing whole gland without abnormal features |
| Equivocal | Less than three abnormal branches |
| Mild | More than three abnormal branches |
| Moderate | Abnormal main duct and branches |
| Marked | As above with one or more of:  Large cavities (>10mm)  Gross gland enlargement (>2×N)  Intraductal filling defects or calculi  Duct obstruction, stricture or gross irregularity  Contiguous organ invasion |

†ERP, endoscopic retrograde pancreatography

**Supplementary Table 2. Rosemont classification for chronic pancreatitis**

| **EUS**^†^ **findings** | **Major criteria** |
| --- | --- |
| 【Parenchymal features】 |  |
| Hyperechoic　foci　with　shadowing | Major A |
| Lobularity　with　honeycombing | Major B |
| Lobularity　without　honeycombing | Minor |
| Hyperechoic　foci　without　shadowing | Minor |
| Cysts | Minor |
| Strands | Minor |
| 【Ductal features】 |  |
| MPD^‡^　calculi | Major A |
| MPD^‡^　contour | Minor |
| Dilated　side　branches | Minor |
| MPD^‡^　dilation | Minor |
| Hyperechoic　MPD^‡^　margin | Minor |

†EUS, endoscopic ultrasonography;‡MPD, main pancreatic duct
